# Brain Responses to Musical Feature Changes in Adolescent Cochlear Implant Users

**DOI:** 10.3389/fnhum.2015.00007

**Published:** 2015-02-06

**Authors:** Bjørn Petersen, Ethan Weed, Pascale Sandmann, Elvira Brattico, Mads Hansen, Stine Derdau Sørensen, Peter Vuust

**Affiliations:** ^1^Center for Functionally Integrative Neuroscience, Aarhus University Hospital, Aarhus, Denmark; ^2^Royal Academy of Music, Aarhus, Denmark; ^3^Department of Aesthetics and Communication – Linguistics, Aarhus University, Aarhus, Denmark; ^4^Central Auditory Diagnostics Lab, Department of Neurology, Cluster of Excellence “Hearing4all”, Hannover Medical School, Hannover, Germany; ^5^Brain and Mind Laboratory, Department of Biomedical Engineering and Computational Science, Aalto University, Aalto, Finland; ^6^Cognitive Brain Research Unit, Institute of Behavioral Sciences, University of Helsinki, Helsinki, Finland; ^7^Department of Psychology and Behavioural Sciences, Aarhus University, Aarhus, Denmark

**Keywords:** cochlear implants, adolescents, music perception, mismatch negativity, music training, rehabilitation, auditory cortex

## Abstract

Cochlear implants (CIs) are primarily designed to assist deaf individuals in perception of speech, although possibilities for music fruition have also been documented. Previous studies have indicated the existence of neural correlates of residual music skills in postlingually deaf adults and children. However, little is known about the behavioral and neural correlates of music perception in the new generation of prelingually deaf adolescents who grew up with CIs. With electroencephalography (EEG), we recorded the mismatch negativity (MMN) of the auditory event-related potential to changes in musical features in adolescent CI users and in normal-hearing (NH) age mates. EEG recordings and behavioral testing were carried out before (T1) and after (T2) a 2-week music training program for the CI users and in two sessions equally separated in time for NH controls. We found significant MMNs in adolescent CI users for deviations in timbre, intensity, and rhythm, indicating residual neural prerequisites for musical feature processing. By contrast, only one of the two pitch deviants elicited an MMN in CI users. This pitch discrimination deficit was supported by behavioral measures, in which CI users scored significantly below the NH level. Overall, MMN amplitudes were significantly smaller in CI users than in NH controls, suggesting poorer music discrimination ability. Despite compliance from the CI participants, we found no effect of the music training, likely resulting from the brevity of the program. This is the first study showing significant brain responses to musical feature changes in prelingually deaf adolescent CI users and their associations with behavioral measures, implying neural predispositions for at least some aspects of music processing. Future studies should test any beneficial effects of a longer lasting music intervention in adolescent CI users.

## Introduction

The cochlear implant (CI) is a neural prosthesis that provides profoundly deaf individuals with the opportunity to gain or regain the sense of hearing. The implant transforms acoustic signals into electric impulses, which are delivered to an electrode array implanted within the cochlea. The electrodes stimulate intact auditory nerve fibers at different places in the cochlea, thus mimicking the tonotopic organization of the healthy cochlea (Loizou, 1999; McDermott, 2004). The clinical impact of the device is extraordinary, allowing postlingually deafened adults to restore speech comprehension and children to acquire language. Adults with prelingual hearing loss may achieve some auditory alerting functions, but rarely speech comprehension (e.g., Petersen et al., 2013a).

The majority of postlingually deafened adult CI users achieve good speech perception in quiet but their perception of music remains poor. Several studies show that due to low spectral resolution and compromised temporal fine-structure information, discrimination of pitch, melody, timbre, and emotional prosody is significantly poorer in CI users than in normal-hearing (NH) listeners (Leal et al., 2003; Kong et al., 2004; Gfeller et al., 2005, 2007; Olszewski et al., 2005; Cooper et al., 2008; Timm et al., 2012; Agrawal, 2013). Nevertheless, there are examples of CI users who seem to enjoy music after repeated listening (Gfeller and Lansing, 1991; Gfeller et al., 2005) and some studies show significantly improved music discrimination after computer-assisted training (Gfeller et al., 2000a, 2002b; Galvin et al., 2007) and after long-term one-to-one musical ear training (Petersen et al., 2012). These findings suggest that CI users typically do not extract all of the (degraded) information available from the CI signal (Moore and Shannon, 2009) and that targeted auditory training maximizes the benefits of the implant (Fu and Galvin, 2008). Beyond the potential beneficial effects on music enjoyment and social functioning, improved music perception may have positive implications for the quality of life in CI users (Gfeller et al., 2000b; Drennan and Rubinstein, 2008; Lassaletta et al., 2008; Wright and Uchanski, 2012; Petersen et al., 2013b). Furthermore, musical training might transfer to non-musical domains and may have beneficial effects on speech perception in noisy surroundings (Qin and Oxenham, 2003; Parbery-Clark et al., 2009; Won et al., 2010) and on the ability to recognize gender and identity of the speaker (Vongphoe and Zeng, 2005).

In this context, the new generation of prelingually deaf children, who have grown up with the assistance of CIs and who have now become teenagers, is of particular interest. While postlingually deafened CI users rely on auditory development formed by previous hearing experience in processing auditory information from the CI, most current adolescent CI users are congenitally deaf and have only heard sound through their implant. In addition, most young CI users were not diagnosed until they were 2–3 years old and subsequently received their CI after the first 3–5 years of life, that is, beyond the sensitive period for cochlear implantation (Sharma et al., 2002b; Kral and Sharma, 2012).

Initially, cochlear implantation was offered primarily to adults, whereas children were included in CI-programs at a later stage (in Denmark since 1993) and only in moderate numbers. Thus, information about this new population of CI users, their educational placement, and linguistic development has so far been sparse. A recent Danish survey indicate that a majority of young CI users communicate by auditory methods (36%) or auditory methods supported by lip-reading (47%), whereas as few as 5% depend on sign language. Background noise, small talk, slang language, joking, irony, and phone conversation with strangers, however, are reported to represent very challenging daily communicative situations (Rosenmeier and Møller Hansen, 2013). While the findings are an encouraging indication of the overall success of pediatric cochlear implantation (Bosco, 2012), these difficulties highlight the need for continuing specialist teaching throughout adolescence (Archbold et al., 2008; Geers et al., 2008; Harris and Terlektsi, 2011). Adolescence is an age when self-identify is forming and social relations, including music listening and preferences, are particularly important in the life of a teenager (North et al., 2000). Considering that well-functioning communicational skills are crucial for adolescent CI users’ well-being, self-esteem, social functioning, and educational prospects (Hansen, 2012), it is pivotal to understand the neural substrates of their speech and music processing to further develop their hearing and speech skills. Nevertheless, while a few behavioral studies have been conducted on adolescent CI users who were prelingually deaf (Geers et al., 2008; Gfeller et al., 2012), no information is currently at hand concerning the neural correlates of musical sound perception and musical training in adolescent CI users.

Auditory processing in CI users can be studied by recording auditory event-related potentials (ERP) using electroencephalography (EEG) (Sharma et al., 2002a; Pantev et al., 2006; Debener, 2008; Sandmann et al., 2009, 2014). One component of the auditory ERP is the mismatch negativity (MMN), which is related to change in different sound features such as pitch, timbre, harmony, intensity, and rhythm (Näätänen et al., 2001, 2007). In contrast to subjective behavioral measures, the MMN represents a reliable and objective marker for CI users’ ability to accurately discriminate auditory stimuli (Sandmann et al., 2010; Torppa et al., 2012) typically elicited pre-attentively, in the absence of participants’ attention toward the stimuli. MMN latency and amplitude reflect the magnitude of perceptual difference between deviant and standard stimulus and are associated with auditory behavioral measures (Näätänen et al., 2007).

A few MMN studies have investigated auditory brain processing of music in children and adult CI users. For instance, Koelsch (2004) reported timbre-evoked MMN responses with reduced amplitudes in postlingually deaf CI users compared to NH control participants. In a study with postlingually deaf adult CI recipients, Sandmann et al. (2010) reported smaller MMN amplitudes for frequency and intensity deviations in CI users compared to NH controls, and found no robust MMNs to duration deviants in neither of the two groups. In a study with early-implanted CI children (mean age 6 years, 10 months), Torppa et al. (2012) reported comparable magnitudes and latencies of MMN responses to three and seven semitone pitch changes in CI and NH children, and significant MMNs to timbre only for a change from piano to cymbal in both groups. Interestingly, Torppa et al. (2014) in a recent longitudinal study found enhanced development of P3a (attention toward salient sounds) to pitch, timbre, and rhythm changes in CI children who sang regularly, not observed in CI children who did not sing.

Using a newly developed musical multi-feature paradigm, Timm et al. (2014) found distinct MMN responses to pitch, timbre, and intensity, but not to rhythm in postlingually deafened adults with CI. In the present study, we wished to study for the first time the neural prerequisites for music perception, and particularly for musical feature change discrimination, in prelingually deaf adolescent CI users by applying the same paradigm as in Timm et al. (2014). We hypothesized that if any MMN would be found to musical feature changes it would testify the existence of neural predispositions for musical feature processing even in prelingually deaf CI users who were not exposed to any musical (or speech) sounds during the critical period of development. Additionally, we wanted to test whether these adolescent CI users would have any beneficial effect even from a short but intensive music training program. For this purpose, the CI users were measured before and after a musical intervention lasting 2 weeks (20 h), consisting of singing, rhythm, and ear training as well as computer-assisted musical quizzes. We predicted that adolescent CI users would show MMNs, which would differ from those of NH peers, particularly with smaller MMN amplitudes and longer latencies to changes in the acoustic properties of musical sounds, reflecting their impaired musical skills as in behavioral tests. Moreover, we expected to observe a relation between the behavioral effects of music training and the MMN amplitude and latency.

## Materials and Methods

### Participants

The participants were all recruited from Frijsenborg Efterskole (post-school) in the city of Hammel, Denmark. Frijsenborg Efterskole has specialized in teaching hearing-aid (HA) and CI users and employs teachers who are specialized in teaching hearing-impaired pupils and provides modern aids that promote teaching and communication, such as multi-frequency FM equipment. The hearing-impaired pupils make up 25% of the students. The remaining part of the pupils is typical NH age mates.

The participants were recruited through a procedure in which they received oral as well as written information about the project. Since all participants, except one, were below the age of 18 years, their parents received written information also and were required to give informed consent on behalf of their children. The participants received no monetary compensation for their time. The study was conducted in accordance with the Helsinki declaration and approved by the Research Ethics Committee of the Central Denmark Region and is part of a broader study.

All of the school’s 12 adolescent CI users signed up for the study, but, due to illness, one had to withdraw from the project. The remaining 11 CI users (6 girls, 5 boys, *M*_age_ = 17.0 years, age range: 15.6–18.8 years), committed themselves to 2 weeks of music training and two sessions of EEG recording and behavioral tests – one before and one after the training period. In the following, T1 and T2 refer to EEG recordings and behavioral tests administered before and after the 2-weeks intervention period, respectively.

The CI participants had a severe-profound/profound congenital or prelingual hearing loss and had received their CI at different points of time in childhood or adolescence (*M*_age_ at implant = 7.5 years; range: 2.2–14.9 years) between 1997 and 2011, with the majority of participants implanted between 2001 and 2003. The mean implant experience was 9.5 years (range: 1.8–15.2). Nine CI users had bilateral implants, in all cases received sequentially (*M*_age_ at implant 2 = 12.0 years; range: 10.5–16.6 years; mean experience w. CI 2 = 5.2; range: 0.1–6.2) and two CI participants had unilateral implants combined with a contra-lateral HA. All of the participants used the Nucleus Freedom device from Cochlear Corporation. All CI participants had NH, monolingual Danish-speaking parents. The clinical and demographic data of the 11 CI participants are shown in Table 1.

**Table 1:** **Clinical and demographic data of the 11 participants in the CI group**.

Participant (gender)	Age at project start (years)	Etiology of deafness	Side of first implant	Contralateral use of HA	CI 1 experience (years)	CI 2 experience (years)	Use of sign-language^e^	Use of lip-reading^e^	Ability to speak on the phone
**CI GROUP**									
CI 1 (F)	17.8	^a^Cong. non-spec.	L		10.1	5.9	4	5	X
CI 2 (F)	15.5	^b^Pendred	R	X	4.1		1	2	X
CI 3 (F)	16.5	Unknown	L		11.1	5.6	5	5	X
CI 4 (M)	16.6	^c^CMV	L	X	3.0		1	2	X
CI 5 (M)	18.8	Cong. non-spec.	R		9.9	5.7	4	2	
CI 6 (M)	17.3	Cong. non-spec.	R		11.4	6.1	3	4	X
CI 7 (F)	16.2	Pendred	R		11.8	5.0	3	3	
CI 8 (M)	16.6	Meningitis	L		13.4	6.0	3	2	X
CI 9 (F)	17.4	^d^Her. non-spec.	R		15.7	6.2	4	3	X
CI 10 (M)	16.7	CMV	L		1.8	0.1	3	5	X
CI 11 (F)	17.6	Cong. non-spec.	L		12.0	6.1	5	5	X
Mean	17.0				9.5	5.2	3.3	3.5	
Range	(15.6–18.8)				(1.8–15.2)	(0.1–6.2)			

*^a^Non-specified congenital hearing loss*.

*^b^Pendred Syndrome*.

*^c^Cytomegalovirus*.

*^d^Non-specified hereditary hearing loss*.

^e^Indicated on a scale where 5 is “everyday” and 1 is “never.”

The NH reference group consisted of 10 participants (2 girls, 8 boys; *M*_age_ = 16.2 years, age range: 15.3–17.0 years), who committed themselves to two sessions of EEG recording and tests with a 14-day-interval. The NH reference group followed their normal school schedule during the project and received no musical training. By testing the NH participants twice, we acquired measurements that could be used for direct comparisons with the CI group before and after training.

#### Musical background

To account for past and recent musical training and experience, the participants filled out a questionnaire concerning their musical background. All NH participants had attended music classes in primary school, as had all CI participants except one. Four CI participants had sung in a choir, which was only the case for two in the NH group. Two in each group stated that they had played in a band at some point. Four CI users had received musical instrument lessons, which was also the case for five NH participants, typically guitar, bass, or drums and in all cases for a short period of time. Based on this information, we judged the musical background in the two groups to be comparable.

### The music training program

The music training program aimed at strengthening the participants’ perception of the fundamental resources in music: pitch, rhythm, and timbre in a combination of active music-making sessions and computer-based listening exercises. The active training part totaled 20 h, scheduled over 6 days, and distributed over 2 weeks. The activities were formed by three elements: rhythm training, singing, and ear training and were led by two masters’ students from Royal Academy of Music, Aarhus and the first author, who has previous experience with music training of adult and pediatric CI users (Petersen et al., 2011, 2012). Training took place in the school’s two music classrooms, which were acoustically well suited and well equipped.

#### Rhythm training

The intention of the rhythm training sessions was to establish a fundamental sense of meter, period, and subdivision in a motivating and physically engaging manner. The sessions involved recurrent exercises including coordination of foot stomping, clapping, and “rapping”. All exercises were in 4/4-time in tempos between 80 and 110 BPM. The exercises were performed in a circle, standing up.

#### Singing

The purpose of the singing training was to establish a sense of basic musical attributes such as high/low, up/down, far/close, and melodic direction. The singing training involved technical instructions about breath control/belly support and exercises, such as glissando (up/down), and imitation of short phrases with focus on long/short, strong/weak, and open/closed vowel sounds in different vocal registers.

#### Ear training

The ear training part aimed at improving the participants’ general music perception skills, particularly timbre, pitch, and melody in a standard classroom setting. The group was introduced to different instruments in live demonstrations. For perception of pitch and melody, the participants were required to identify the direction of two notes (up, down) or three notes (up-down, down-up) or recognize familiar melodies presented on piano or other instruments.

#### Musical quizzes

To support the ear training sessions, several computer applications, presented as musical quizzes, were developed and made available through download from a website. The quizzes were adapted and expanded versions of applications described in Petersen et al. (2012), aiming to train discrimination of melodic contour, timbre, melody, and rhythm. All quizzes were designed with a familiarization part followed by a number of trials, which required the user to match presented sounds with corresponding icons on the screen. The participants were asked to train everyday for 10–20 min during the 2-weeks training period.

### EEG recording

#### Stimuli and procedure

Electroencephalography was recorded with a musical multi-feature MMN paradigm (Vuust et al., 2011), in a version previously adapted for a study with adult CI users (Timm et al., 2014). The musical multi-feature paradigm presents musical standards, pseudorandomly violated by different deviants in the context of musical four-tone patterns. The four-tone patterns consist of major triads arranged in an “Alberti bass” configuration, an accompaniment commonly used in the Western musical culture.

**Figure 1:**
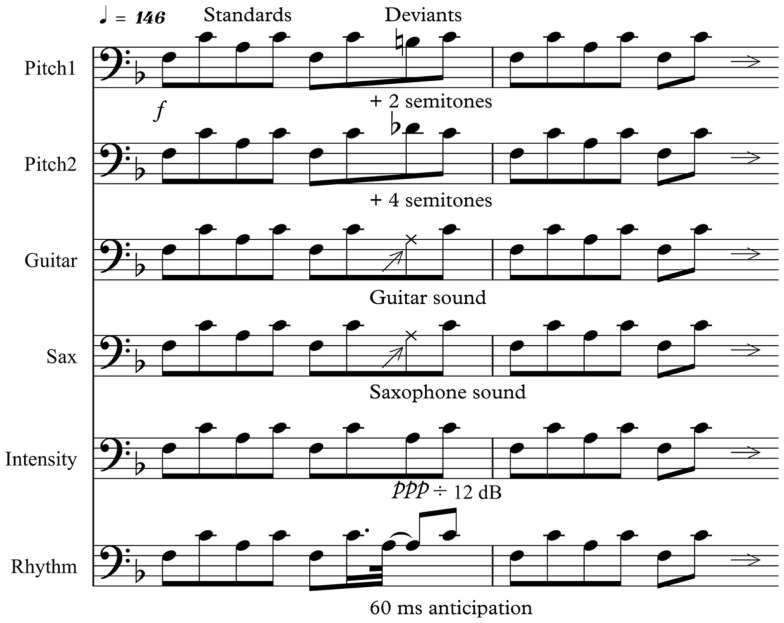
**“Alberti bass” patterns alternating between standard sequence played with piano sounds and a deviant, here in the key of F**. Deviants were introduced randomly and patterns were pseudorandomly transposed to the keys of G, A, or C with an interval of six bars. Each tone was 200 ms in duration, with an ISI of 5 ms, yielding a tempo of approximately 146 beats/min. Comparisons were made between the third note of the standard sequence and the third note of the deviant sequence.

In the adapted configuration, deviant patterns were similar to standards, except that the third tone of the pattern was exchanged with one of six deviants: (1) pitch deviant (Pitch1_D1_), which was created by raising the standard note by two semitones, (2) pitch deviant (Pitch2_D2_), which was created by raising the standard by four semitones, (3) timbre deviant (Gui_D3_), which was created by replacing the standard piano timbre with the sound of an electric guitar, (4) timbre deviant (Sax_D4_), which was created by replacing the standard piano timbre with the sound of a saxophone, (5) intensity deviant (Int_D5_), which was created by reducing the original intensity by 12 dB, and (6) rhythm deviant (Rhy_D6_), which was created by anticipating the third note by 60 ms. In contrast to the more subtle deviants encompassed in the original multi-feature paradigm aimed at musicians and non-musicians (Vuust et al., 2011), the deviants in the present study were enhanced, thus taking the crude sound representation of the CI into consideration. Each tone was in stereo, 44,100 in sample frequency, and 200 ms in duration, having an inter-stimulus-interval (ISI) of 5 ms. For the Rhy_D6_ deviant, the note prior to the third note was shortened to 140 ms and the ISI between third and fourth note extended to 65 ms. The position of the fourth note was preserved, thus leaving the metric pulse uninterrupted. To make the stimuli more musically interesting, we changed the key every sixth measure, allowing for the six different types of deviants to appear in four different keys. The order of the four possible keys (F, G, A, and C) was pseudo-randomized, so that each key appeared six times in the duration of the paradigm. The keys were kept in the middle register of the piano with the bass note between F3 and C4. The stimuli were presented in Presentation software (Neurobehavioral Systems). The paradigm presented a total of 4608 stimuli, making the duration of whole experiment approximately 18 min, including two 1-min-pauses (Figure 1). For more details about the paradigm, see Timm et al. (2014).

#### EEG data recording and analysis

Recording of EEG took place in an acoustically dampened room at Frijsenborg Efterskole. Participants were seated in front of two active loudspeakers (Genelec 8020B; Genelec Oy, Iisalmi, Finland) placed to their left and right side with a 45° angle, approximately 0.5 m distance from the participants’ ear. Participants were instructed to ignore the auditory stimuli and watch an animated subtitled movie presented without sound.

The stimuli were presented at 65 dB SPL. CI users used their everyday processor settings during the EEG session. To assure the most comfortable level, participants were exposed to the stimuli briefly before the EEG recording, thus getting an opportunity to adjust their processor settings. To assure comparable conditions for CI participants, bilateral CI users were asked to use only their preferred implant and bimodally aided participants were asked to remove their hearing aid.

Electroencephalography was recorded from 30 Ag/AgCl electrodes placed according to the International 10–20 system and using a BrainAmp amplifier system (Brainproducts, Gilching, Germany). Two additional electrodes were placed below the left and right eye to record the electrooculogram. For CI users, some channels could not be used because of the location of the CI device. Data were recorded with a sampling rate of 500 Hz using the position FCz as reference, and were analog filtered between 0.02 and 250 Hz. Electrode impedances were maintained below 5 kΩ prior to data acquisition.

Electroencephalography data were analyzed with custom scripts and EEGLAB 12.0.2.4b (Delorme, 2004) running in the MATLAB environment (Mathworks, Natick, MA, USA). The preprocessing was done using a two-step procedure, optimized for artifact correction with independent component analysis (ICA) (e.g., Debener et al., 2010). In the first step, the raw data were offline filtered (1–40 Hz) and epoched into continuous 2 s intervals. Intervals containing unique, non-stereotyped artifacts were rejected (threshold: 3 SD). Infomax ICA was computed on the remaining data. In the second step, the resulting ICA weights were applied to the raw data filtered between 0.5 and 30 Hz. Note that the different filter settings for ICA training and ERP analysis was done according to previous recommendations (Debener et al., 2010) and accounted for the otherwise adverse effect of slow amplitude drifts (<1 Hz) on ICA data decomposition. Independent components representing eye-blinks, horizontal eye movement, and electrocardiographic artifacts were identified semi-automatically and were corrected from all datasets using CORRMAP (Viola et al., 2009). Next, the data were segmented from -100 ms to 400 ms relative to stimulus onset, and components representing CI artifacts and other non-cerebral activity were identified by visual inspection of various component properties. Independent components representing CI artifacts were identified by the centroid on the side of the implanted device, and by the time course of component activity (for details on the reduction of CI artifacts by means of ICA, see Gilley, 2006; Debener, 2008; Sandmann et al., 2009). The total number of rejected ICA components was (means and SEM): 8 ± 0.7 for the CI users before training, 9 ± 0.7 for the CI users after training, 10 ± 0.7 for the NH listeners in the first session, and 9 ± 0.9 for the NH listeners in the second session. The data were then pruned of unique, non-stereotyped artifacts (threshold: 3 standard deviations), and unused channels were interpolated (mean: 2 electrodes; SEM: 0.4; range: 1–3 electrodes) using the EEGLAB function eeg_interp.m, before re-referencing the data to a common average reference. Finally, ERPs were obtained by time-domain averaging, and the pre-stimulus interval from −100 to 0 ms was used for baseline correction.

#### MMN quantification

Difference waveforms were computed for each participant by subtracting the response to the standard stimulus from each of the six deviant stimuli. MMN’s were identified with the following procedure. First, a grand-average difference wave was constructed for each deviant by combining the difference waves from the two recording sessions. This was done separately for the NH and the CI group. Next, a 40 ms time window was defined, centered on the most negative point at 75–205 ms in the grand-average difference waves. Finally, the MMN was measured as the peak amplitude within the 40 ms window at the Fz electrode site for each participant, deviant type, and recording session. To avoid erroneously high or low values, three data points on either side of the peak were included in the peak measurement (14 ms duration in total). MMN latency was measured as the peak amplitude between 75 and 205 ms at Fz electrode for each participant, deviant type, and recording session.

### Behavioral measurements

#### Musical multi feature discrimination task

All participants completed a music discrimination test before and after the intervention period. The purpose was to obtain a behavioral measurement of auditory discrimination accuracy of the six musical deviants also used in the MMN paradigm. The test was designed as a three-alternative forced-choice task (3-AFC), in which the participants were presented with a similar four-tone piano pattern as used in the EEG experiment, restricted, though, to the key of C major. The pattern was presented thrice in a row, twice in the standard, and once in the deviant condition. The deviant patterns were presented equally often and were repeated 6 times in random order, occurring as either the first, the second, or the third pattern, adding to a total of 36 trials. Participants were instructed to click pictorial representations of the pattern, indicating at which position the deviating pattern had occurred. The scores were converted to percent correct hit rates for the six deviant conditions.

##### Dantale II test

To measure speech comprehension, we used the Danish speech material Dantale II (Wagener et al., 2003). In the applied configuration, this sentence test adapts to the respondent’s performance by increasing or decreasing the volume of the speech, holding the background noise at a constant level. The result of the test is given as the speech reception threshold (SRT) in this case the signal-to-noise ratio for 50% word intelligibility. The participants completed three lists, one training list and two trial lists, thus testing perception of 100 words in total. All participants listened through headphones, as did the test administrator. Bilateral CI users were allowed to use both CIs, whereas bimodally aided users were required to switch off their HA but keep it plugged. This measure was taken to secure that conditions were as comparable as possible and to exclude any assistance from potential residual hearing. CI users as well as NH participants completed the test at both recording sessions (T1 and T2). The rationale for testing NH participants twice was first to identify any effects of time and, second, to identify learning effects, which have been reported previously (Pedersen and Juhl, 2013).

### Statistical methods

#### MMN responses

In a first step, we tested for significant MMN amplitudes by performing two-tailed one-sample *t*-tests on each of the deviant difference waves using the ttest.m function in Matlab (Mathworks, Natick, MA, USA). Following this, similar to previous MMN studies on CI users (Sandmann et al., 2010; Timm et al., 2014), we tested for main effects of group, time, and deviant type, and possible interactions between these effects by performing mixed-effects ANOVAs separately on MMN amplitudes and latencies with the between-subjects factor Group (NH and CI) and the within-subjects factors Time (T1 and T2) and deviant type (1–6). *Post hoc* tests were performed using Bonferroni-corrected *t*-tests.

#### Behavioral tests

The analysis of the behavioral data from the musical multi-feature discrimination test was performed in a separate mixed-effects ANOVA with the between-subjects factor of Group (NH and CI) and the within-subjects factors of time (T1 and T2) and deviant type (1–6).

To identify significant training effects and group differences as measured by the Dantale II test, we analyzed the SRT values using independent (between groups) and paired (within groups) *t*-tests.

Correlation analyses between EEG results, behavioral results, and clinical data were done using Spearman’s product–moment test. For all tests, the level for significance was set at 0.05, and the significant results are reported. All tests were performed in SPSS (IBM SPSS Statistics for Windows, Version 21.0. Armonk, NY, USA: IBM Corp.).

## Results

### MMN amplitudes

**Figure 2:**
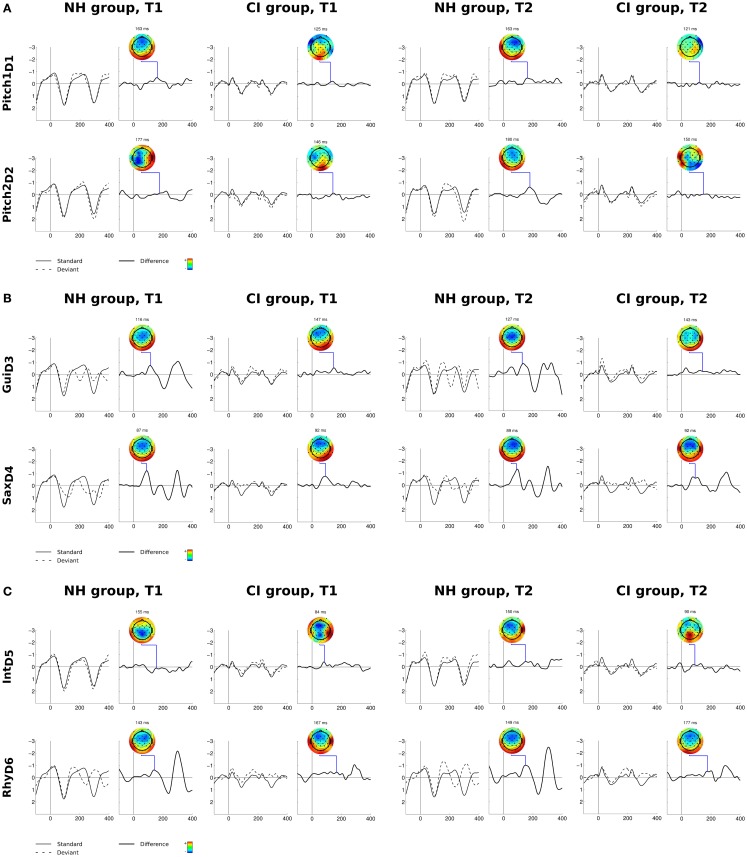
**(A–C)** Grand-average ERPs and EEG voltage isopotential maps for six types of deviants (vertical) in the two experimental groups at T1 (left) and T2 (right). For each deviant, left panels show responses to the standard (solid line) and to the deviant (dotted line). Right panels show difference waves. Isopotential maps illustrate the difference between the responses to deviants and standards averaged in an interval of ±3 ms around maximal peak amplitudes. X-axis values are in milliseconds; Y-axis values are in microvolts.

For the CI users, the musical multi-feature paradigm elicited significant MMNs for deviants Gui_D3_, Sax_D4_, Int_D5_, and Rhy_D6_ at both T1 and T2. For the two pitch deviants, the CI users exhibited a significant MMN only for Pitch1_D1_ and only at T1. For the NH listeners, our analyses showed significant MMNs for all six deviants at both times of testing, except for the T1 Int_D5_ (Figures 2A–C; Tables 2 and 3).

**Table 2:** **Amplitudes and latencies of the MMN in response to different musical features in CI users at T1 and T2**.

CI users		T1 results				T2 results			
Deviant	Interval (ms)	Peak amplitude (μV)	*t*	SD	Latency (ms) (SD)	Peak amplitude (μV)	*t*	SD	Latency (ms) (SD)
Pitch1_D1_	103–143	−0.45	−3.49**	0.43	125 (11.8)	−0.27	−1.54	0.58	121 (9.7)
Pitch2_D2_	128–168	−0.19	−1.18	0.55	146 (10.2)	−0.22	−1.72	0.42	150 (11.7)
Gui_D3_	125–165	−0.63	−6.41**	0.33	147 (8.8)	−0.45	−3.80**	0.39	143 (11.8)
Sax_D4_	72–112	−0.88	−6.06**	0.51	92 (11.0)	−0.88	−6.55**	0.44	92 (12.7)
Int_D5_	67–107	−0.42	−3.10*	0.45	84 (8.0)	−0.36	−2.34*	0.51	90 (11.6)
Rhy_D6_	152–192	−0.57	−5.24**	0.36	167 (10.2)	−0.63	−4.62**	0.45	177 (7.2)

(*p = 0.01; **p < 0.001).

**Table 3:** **Amplitudes and latencies of the MMN in response to different musical features in normal-hearing controls at T1 and T2**.

NH participants		T1 results				T2 results			
Deviant	Interval (ms)	Peak amplitude (μV)	*t*	SD	Latency (ms) (SD)	Peak amplitude (μV)	*t*	SD	Latency (ms) (SD)
Pitch1_D1_	143–183	−0.68	−5.48**	0.39	163 (11.6)	−0.57	−6.73**	0.27	163 (8.3)
Pitch2_D2_	158–198	−0.39	−3.45**	0.36	177 (12.6)	−0.76	−5.05**	0.47	180 (10.2)
Gui_D3_	101–141	−0.86	−4.61**	0.59	116 (10.4)	−1.13	−5.32**	0.67	127 (11.4)
Sax_D4_	68–108	−1.30	−6.94**	0.59	87 (7.2)	−1.41	−5.62**	0.79	89 (7.8)
Int_D5_	132–172	−0.34	−1.98	0.54	155 (9.6)	−0.42	−5.94**	0.22	150 (12.1)
Rhy_D6_	126–166	−0.72	−5.24**	0.43	143 (11.1)	−1.11	−15.56**	0.22	149 (9.4)

*p = 0.01; **p < 0.001.

Our mixed-effects analysis of the MMN amplitudes showed a significant main effect of Group, *F*(1, 19) = 8.43; *p* = 0.009, driven by overall smaller MMN mean amplitudes in the CI users compared to the NH participants (mean value for combined MMNs across all deviants: CI users: T1: −0.54 μV, SD: 0.49, T2 −0.47 μV SD: 0.58; NH controls: T1 −0.66 μV, SD: 0.61, T2 −0.94 μV, SD: 0.58).). Furthermore, we found a significant main effect of deviant type [*F*(5, 95) = 15.77; *p* < 0.001], predominantly deriving from significantly larger amplitudes elicited by the Sax_D4_ compared to the other five deviants. There was also a significant interaction between Group and Time [*F*(1, 19) = 7.3; *p* = 0.014] driven by a significantly larger overall MMN negativity in the NH group at T2 compared to the CI group (*p* = 0.002; NH: −0.94 μV; CI: −0.47 μV). The *post hoc* comparison of the two groups at T1 was not significant. The Group by Deviant Type interaction was non-significant. Also, the three-way interaction Group × Time × Deviant Type was non-significant. Explorative *t*-tests showed a significant difference between the MMN amplitudes of the two groups for Pitch1_D1_ [*t*(1, 19) = −2.53; *p* = 0.02], Gui_D3_ [*t*(1, 19) = −2.32; *p* < 0.037], and Rhy_D6_ [*t*(1.19) = −2,38; *p* < 0.028], in each case driven by larger mean amplitudes in the NH participants compared to CI users.

### MMN latencies

The mixed-effects analysis on MMN latencies showed a significant main effect of Group, *F*(1, 19) = 83.55; *p* < 0.001, driven by overall shorter MMN mean latencies in CI users than in the NH participants (mean value for combined MMN latencies: CI users: 127.15, SD: 31.75, NH listeners: 141.97, SD: 31.40). Furthermore, we found a significant main effect of Time [*F*(1, 19) = 5.05; *p* = 0.037], driven by overall longer MMN latencies in both groups at T2 compared to T1 (mean latency difference: 2.43 ms). Finally, we found a significant main effect of Deviant Type, *F*(5, 95) = 258.66, *p* < 0.001 and an interaction between Deviant Type and Group, *F*(5, 95) = 122.6, *p* < 0.001. The three-way interaction Group × Time × Deviant Type was non-significant.

*Post hoc t*-tests for mean latencies across T1 and T2 with respect to Deviant Type showed that for CI users Gui_D3_ and Rhy_D6_ deviants were significantly longer compared with MMN latencies in the NH participants [Gui_D3_, *t*(1, 19) = −5.9; *p* < 0.001; Rhy_D6_, *t*(1, 19) = −8.4, *p* < 0.001]. In contrast, for deviants Pitch1_D1_, Pitch2_D2_, and Int_D5_, we found significantly shorter latencies in the CI users compared to the NH group at T1and T2 [Pitch1_D1_, *t*(1, 19) = 12.58; *p* < 0.001; Pitch2_D2_, *t*(1, 19) = 9.74; *p* < 0.001; Int_D5_, *t*(1, 19) = 20.71, *p* < 0.001] (Figures 2A–C; Tables 2 and 3).

### Behavioral musical multi feature discrimination test

**Figure 3:**
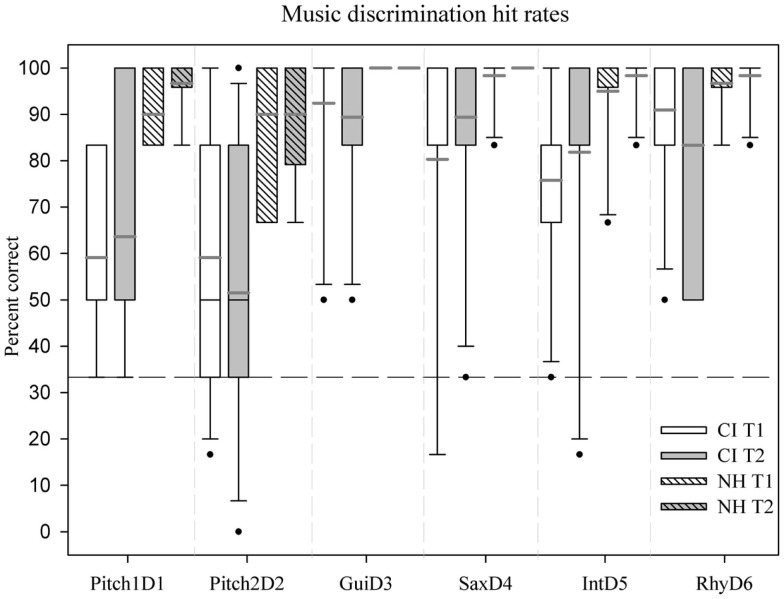
**Box plot showing mean hit rates of the two groups for the six deviants at T1 and T2**. Whiskers (error bars) above and below the box indicate the 90th and 10th percentiles. Solid black line represents the median, gray line represents the mean. Dots represent outlying points. Dashed line represents chance level.

Our mixed-effects analysis showed a significant main effect of Group, *F*(1, 19) = 13.04; *p* = 0.002, driven by an overall 19.72% point lower score in CI users compared with NH participants. Furthermore, the analysis showed an interaction between Deviant Type and Group, *F*(5, 19) = 13.79, *p* = 0.001. According to *post hoc t*-tests, this interaction was driven by significantly lower overall hit rates by the CI users for discrimination of Pitch1_D1_ [*T*(5, 19) = 5.27, *p* = < 0.001], Pitch2_D2_ [*T*(5, 19) = 4.13, *p* = 0.001], Gui_D3_ [*T*(5, 19) = 2.41, *p* = < 0.037], and Int_D5_ [*T*(5, 19) = 2.63, *p* = 0.023] compared to NH controls. The groups did not differ for the Sax_D4_ or Rhy_D6_ deviants (Figure 3). We found no effect of Time.

### Dantale II test

The CI users produced mean speech recognition threshold values of 1.0 at T1 and of 0.04 at T2, indicating a (non-significant) improvement in their ability to recognize speech in background noise. The CI users’ mean SRT values were significantly higher than those of the NH participants at both T1 and T2 (*p* < 0.001) and displayed also a high variability ranging from −3.9 to 10.9 dB SNR.

**Figure 4:**
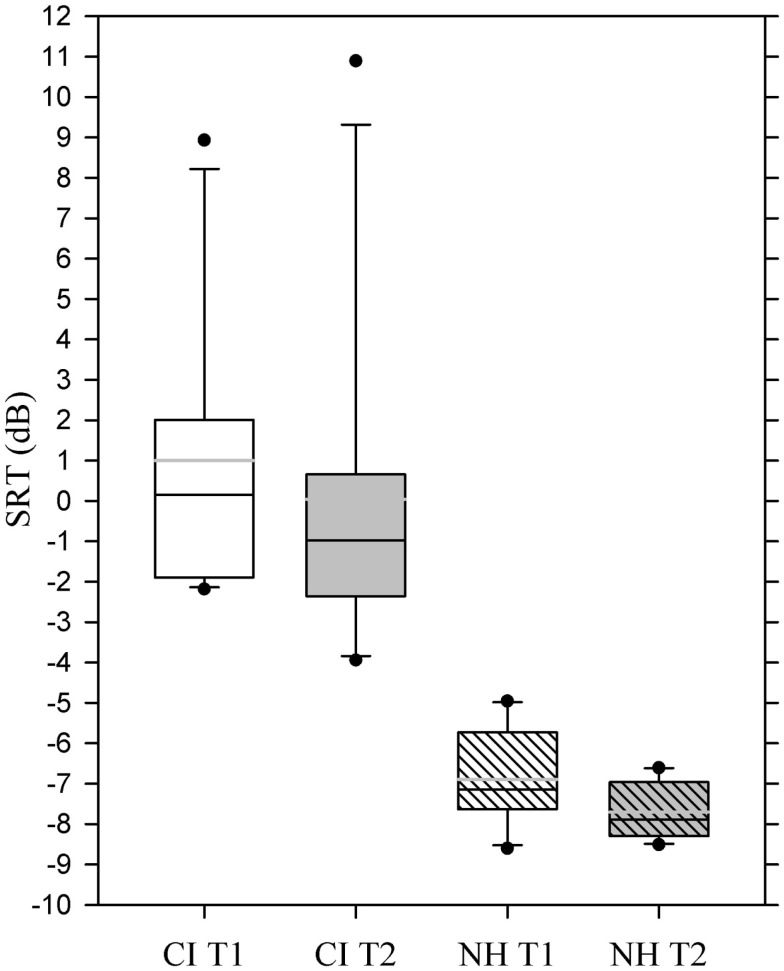
**Box plot showing mean speech recognition thresholds for the two experimental groups at T1 and T2**. Whiskers (error bars) above and below the box indicate the 90th and 10th percentiles. Solid black line represents the median, gray line represents the mean. Dots represent outlying points. Note that a more negative value corresponds to a better performance.

The mean SRT for NH participants was −6.9 at T1 and −7.7 at T2, which represented a significant improvement [*t*(1, 9) = 3.31, *p* = 0.009] (Figure 4).

### Correlations

Correlation analyses were performed for CI users between MMN amplitudes and latencies and behavioral music discrimination scores and Dantale II T2 results and demographic data. Because our ANOVAs showed no main effect of Time, we computed values that were averaged across T1 and T2 for MMN amplitudes and behavioral music discrimination data.

**Figure 5:**
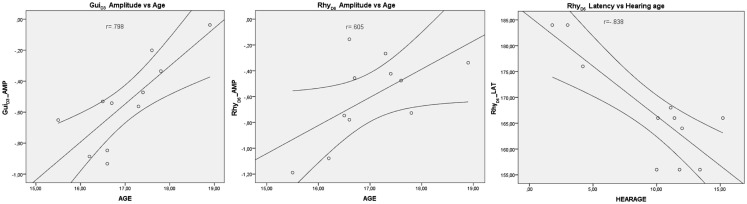
**Scatter plots illustrating the correlation between the mean MMN amplitude to the Gui_D3_ and age (left panel), mean amplitude to the Rhy_D6_ deviant and age (middle panel), and mean MMN latency for the Rhy_D6_ deviant and hearing age (=implant experience) in the adolescent CI users**.

For the MMN data, a significant positive association was found between mean amplitudes for the Gui_D3_ (*r* = 0.798) and Rhy_D6_ (*r* = 0.605) and age, indicating that younger CI users had larger MMN responses than older CI users for these two deviants. Furthermore, we found a significant negative association between hearing age (implant experience) and mean latency for the Rhy_D6_ (*r* = −0.838), indicating that CI users with higher hearing age had MMN responses with shorter latency for this deviant (Figure 5). A similar non-significant association was found for the Sax_D4_ deviant (*r* = −0.592, *p* = 0.055).

Hit rates for behavioral discrimination of the six different musical deviants showed a general positive association with each other. Significant correlations were found between discrimination of IntD5 and Pitch1_D1_ (*r* = 0.699), Gui_D3_ (*r* = 0.642), Sax_D4_ (*r* = 0.907), and Rhy_D6_ (*r* = 0.789) and between Rhy_D6_ and Pitch2_D2_ (*r* = 0.665) and Sax_D4_ (*r* = 0.807). Further associations were found between behavioral discrimination scores and Dantale II SRTs, in all cases, however, driven by an extraordinarily high SRT by a single outlier.

## Discussion

The current study measured behavioral and electrophysiological correlates of music perception in prelingually deaf adolescents before and after a 2-week music training program. A group of age-matched NH listeners served as controls. Overall, the results revealed smaller MMN amplitudes and shorter MMN latencies in CI users than in NH listeners. More specifically, the adolescent CI users showed robust MMN responses for deviations in timbre, intensity, and rhythm. For pitch deviants, we found no consistent MMNs in CI users, which was also reflected in the CI users’ poor hit rates for behavioral pitch discrimination. The findings suggest that even though these adolescents received their implants beyond the optimal age for cochlear implantation (Kral and Sharma, 2012) and have formed their perception of sound solely through the implant, their auditory pathways have been sufficiently developed to allow some discrimination of details in music, predominantly within timbre, timing, and intensity. The study complements previous MMN studies with adult and pediatric CI users (Sandmann et al., 2010; Zhang, 2011; Torppa et al., 2012, 2014), showing potential ability also in prelingually deaf, late-implanted adolescent CI users to process features of music, even when embedded in a complex auditory context.

Consistent with our hypothesis, we found significantly diminished overall amplitudes in the CI users compared to NH controls. The difference, however, reflected differential responses depending on deviant type, with smaller MMN amplitudes elicited by the Pitch1_D1_, Gui_D3_, and Rhy_D6_ deviants and comparable amplitudes elicited by the Sax_D4_ and Int_D5_ deviants. In line with this, we found significantly poorer overall behavioral discrimination scores, which confirm that MMN responses for changes in various kinds of stimuli are reflected in discrimination accuracy (Näätänen et al., 2007). Contrary to our hypothesis, we found significantly shorter overall MMN latencies in the CI users compared to NH peers. Again the difference was linked to deviant type; Gui_D3_ and Rhy_D6_ deviants showed significantly longer latencies, whereas the Int_D5_ and the two pitch deviants were elicited significantly earlier than those of the NH reference. Latencies for pitch, however, should be judged with caution, given the fact that the pitch MMNs were non-significant for Pitch1_D1_ at T2 and for Pitch2_D2_ at both time points.

### Music training

For most of the young CI users, this project was their first experience with structured and targeted music making and certainly challenging. Indeed, they generally responded with great enthusiasm to the different exercises and tasks and also displayed a marked progress in their musical competences. Nevertheless, in contrast to our hypothesis, we were unable to observe any progress in the young CI users’ discrimination skills at either a neuronal or behavioral level. This lack of progress could be due to the brevity of the program. Moreover, the broad-spectrum and music-making nature of the training may have been insufficiently focused to reliably strengthen the specific auditory skills in demand for the tests in such a short period of time. It is important to emphasize, however, that because of interference with the participants’ school activities, an extended training period was not an option and that the music-making approach was deliberately chosen to ensure maximum appeal to the participants. Evenly important, according to self-report, the CI participants spent much less time training with the musical quizzes than requested. Despite instant feedback and progressive design, the quizzes offered little excitement in comparison with current computer games and may simply have appeared less appealing. Future studies should investigate the possible advantages of applications, preferably for smart phones or tablet computers, which offer auditory training of music discrimination skills in an adaptive, socially interactive, and game-like design (Lee and Hammer, 2011).

Contrary to our predictions, we found an overall progress in MMN amplitude in the NH group, who received no music training. We could speculate that NH subjects show training effects simply by being a second time exposed to the same sound stimulation (Paukkunen, 2011). Instead, CI users, even if they had a musical training, did not show any advantage at T2, probably as a consequence of their deficits in musical sound processing. To be visible, the exposure to sounds in CI users should most likely be very long and intensive, whereas in normal subjects some transient neural effects are observable even already after 20 min of discrimination training (Jäncke et al., 2001; Brattico et al., 2003; Lappe et al., 2011).

### Rhythm

Previous behavioral studies with postlingually deaf CI users have documented that discrimination of complex rhythm is difficult (Leal et al., 2003; Kong et al., 2004; Drennan and Rubinstein, 2008). In that respect, we were encouraged to find that the adolescent CI users were able to produce significant MMN responses to a change in rhythm as fast as 60 ms and produce discrimination scores that were not significantly different from the NH reference. This is an indication of the ability of these young CI users to extract fast temporal information despite prelingual deafness and late implantation, as well as the accuracy with which timing features are transmitted in current CI technology. Ability to discriminate rhythm may assist young CI users in general when listening to music, especially for genres that tend to have strong rhythmic elements paired with lyrics (Gfeller et al., 2012). Moreover, poor perception of rhythm has been associated with poor perception of syllable stress and dyslexia (Overy, 2003; Overy et al., 2003; Huss, 2011), and it is possible that training of rhythm, on a long-term, could form a beneficial part in auditory–oral therapy for young CI users (Looi and She, 2010; Petersen et al., 2012).

Our results are in contrast with Timm et al. (2014) who found no robust MMN response to the rhythm deviant in their adult CI users. The authors speculated that one of the sources to this absence of MMN could possibly be that the relatively small deviation of 60 ms was too difficult to extract, especially when embedded in a complex auditory scene. There may be several sources to the discrepancy between the two studies. First, the CI users in the present study were significantly younger (mean age 17 vs. 43.5 years), which may influence neural processing of auditory stimuli. Second, the adolescent CI users all used the most updated implant device in contrast to the adult CI users’ selection of brands and models, which might result in some differences in timing accuracy. A minor difference in the way the rhythm deviant was presented in the two studies may also have contributed to the different results. In the present study, the position of the fourth note was preserved, thus leaving the metric pulse uninterrupted. In the Timm et al. (2014) study, the position of the fourth note was altered in accordance with the early third note, thereby shifting the metric pulse. Thus, the rhythm deviant in the present study deviates in three ways. First, it cuts the preceding note short, which could be perceived as a deviation of duration. Second, the third note comes early, violating the rhythmic flow and, third, the fourth note comes late, caused by the longer gap between notes 3 and 4. By inspecting the difference wave plots for the rhythm deviant (Figure 2C), it appears that this multifaceted deviation evokes not only a significant MMN in the 143–173 ms window after stimulus onset but also a consistent and even stronger negative peak around 325 ms. This effect is identical and consistent across groups and time points and we speculate that it reflects a second MMN in response to the late fourth note.

### Timbre

Both the guitar and the saxophone deviants elicited significant brain responses in our two experimental groups. This is in contrast to findings by Torppa et al. (2012) who in a study with CI and NH children found significant MMNs only to a large change from piano to cymbal but not to changes from piano to violin or to cembalo. They did, however, find indications of a general improvement with age in the children’s ability to detect changes between instruments, which could partly explain this discrepancy. Our findings are in line with Timm et al. (2014) who found similar strong MMN responses to timbre changes in postlingually deaf adult CI users. Interestingly, in both studies the saxophone deviant showed the largest effect compared to the remaining deviants and amplitude and latency that were not significantly different from those of NH listeners. It should be emphasized, however, that the latency of the MMN for this particular deviant was quite different in the two studies, elicited around 92 ms in the present and around 165 ms in the Timm et al. (2014) study. Since both the stimuli and the experimental settings were identical, we speculate that differences in age may be the primary source of this difference in timing.

As opposed to the saxophone deviant, CI users’ MMN responses to the guitar deviant showed significantly smaller amplitudes and significantly longer latencies than those of NH controls, indicating reduced discrimination accuracy. The neurophysiological findings were reflected in behavioral performance in which the CI users produced discrimination scores, which were comparable to the NH level for the saxophone but not for the guitar deviant. This suggests that the sound of a saxophone, which is characterized by a slow attack and a soft tone, represents a larger deviation from the piano tone than the sharp distinct sound of the guitar. Moreover, in an MMN study, which is based on the theory of predictive coding (Baldeweg, 2006), an unexpected occurrence of a saxophone sound in a stream of piano notes represents not only a change of timbre but also a change in timing and intensity, which could also partly explain the observed difference.

So are adolescent CI users as good or almost as good as NH peers in discrimination of timbre? No, probably not. Discrimination of timbre involves perception of several acoustic parameters, particularly the temporal envelope (rise time, duration, and decay) and harmonic spectrum of a sound, and is usually poor in CI users (Gfeller et al., 2002a; McDermott and Looi, 2004; Drennan and Rubinstein, 2008; Spitzer et al., 2008; Timm et al., 2012). The fact that the adolescent CI users were able to detect changes in timbre does not necessarily mean that they would be able to recognize a musical instrument. It does, however, indicate that they possess some basic prerequisites for developing this skill and that the implant transmits sufficient spectral information to allow detection of changes in timbre (Koelsch, 2004). Previous studies have showed enhanced abilities to discriminate timbre after computer-assisted training (Fujita and Ito, 1999; Leal et al., 2003; Pressnitzer et al., 2005; Driscoll et al., 2009) and long-term individual training (Petersen et al., 2012). Improved perception of timbre may add positively to the esthetic enjoyment of music listening and may also be beneficial in other aspects of listening such as recognition of gender or speaker in auditory-only acoustic communication, which are notoriously challenging with CIs (Vongphoe and Zeng, 2005).

### Pitch

Except for the Pitch1_D1_ deviant at T1, the CI group did not exhibit significant MMN responses to changes in pitch of neither two nor four semitones and produced pitch discrimination scores, which were significantly below the NH level. This pitch discrimination deficit may indicate that the neuronal connections of the auditory pathways were not established in the appropriate time window of opportunity, leaving the potential for developing pitch processing abilities very limited (Sharma et al., 2005; Sharma, 2006). Despite ability to produce significant MMNs for pitch deviants, the adult CI users in the study by Timm et al. (2014) showed significantly diminished amplitudes, longer latencies, and lower hit rates for the two and four semitones pitch deviants compared to NH controls. This indicates that, at least for small pitch change detection, the advantages of postlingually deafened CI users, who rely on auditory skills developed prior to their hearing loss, over prelingually deaf adolescent CI users, whose auditory development is based exclusively on implant experience, may be rather small.

Interestingly, Torppa et al. (2012) in a recent study found magnitude and timing of MMN responses to three and seven semitone changes of pitch in early-implanted CI children that were comparable to those of NH controls. The authors suggested that harmonic components of the presented piano tones may be sufficiently separated in frequency to allow accessibility of spectral cues to a change in pitch to the CI children. While the children in the Torppa et al.’ study had a mean age at switch-on of 21.5 months (range 14–37 m), the adolescents in the present study were implanted significantly later (mean age at switch-on: 7.4 years). We speculate that the delayed stimulation of the auditory system is the primary cause of the poor pitch processing observed in the adolescent CI users. Furthermore, the previous study used a multi-feature MMN paradigm, which presented repeated piano tones in contrast to the present study, which presented deviants in a complex musical context and randomly changing keys.

We observed a significant MMN for the Pitch_D1_ at T1 but not at T2, implying a reverse effect of the training. However, considering the intensive focus on pitch and melody included in both the singing and ear training activities, we hardly believe that is the case. More likely, the inconsistent pitch MMNs reflect the suboptimal recording conditions and possible variability across sessions in participant behavior, which may have prevented the weak pitch responses from passing the statistical thresholds. Alternatively, pitch MMNs were elicited but could not be identified due to overlap by other potentials. Finally, the rather short SOA used here prevented identifying a latency longer than 200 ms. Considering that the NH children showed MMN latencies to pitch deviants close to 200 ms, it may well be that we simply missed it.

### Intensity

Electrical hearing produces a much narrower dynamic range than acoustic hearing (Galvin et al., 2007; Veekmans et al., 2009). We were therefore surprised to find MMN responses to the Int_D5_ deviant, which were not significantly different in amplitude from those of the NH listeners. It should be emphasized, however, that the NH responses were surprisingly weak for these deviant and non-significant at T2, indicating a generally small effect of this deviation. Furthermore, although significantly poorer than the NH reference, the CI users’ hit rates for discrimination of intensity were well above chance. This indicates that despite the limited dynamics of the implant, the 12 dB decrement in intensity is transmitted reliably even in prelingually deaf adolescent CI users. The results are partly consistent with a previous MMN level-study with adult CI users, in which Sandmann et al. (2010) found significant MMN responses to a 12 dB intensity decrement but not to two smaller 4 and 8 dB intensity decrements. Future studies should investigate discrimination of changes of intensity in adolescent CI users in more detail.

While our two experimental groups produced similar but small MMN amplitudes in response to the Int_D5_ deviant, the latencies differed significantly. The MMNs of the CI users peaked around 84 ms while those of the NH listeners peaked around 150 ms. This difference may reflect different processing of this particular deviant. However, as with the MMN responses for pitch, we cannot exclude the possibility that the latency values for intensity in the CI group may reflect activity that is different from the activity reflected in the later peaks among NH participants.

### Speech perception in noise

The marked improvement in the CI users’ SRT s suggested a transfer effect from the music training. The similar and significant progress in the non-trained NH group, however, indicates that these improvements are the results of a test learning effect, as seen in previous studies (Pedersen and Juhl, 2013). The Dantale II test requires the ability to identify words in spoken sentences in background noise and subsequently match these with a matrix of optional words on a computer screen, a complex task that relies on both reading skills and working memory and may benefit from previous exposure. These requirements may also explain the huge variability observed in the CI group reflecting possible differences in the participants’ linguistic and cognitive development (Burkholder and Pisoni, 2003). Naturally, the variance may also reflect other factors such as history of hearing loss and CI functionality. None such predictive factors, however, were identified in our correlational analyses.

### Musical multi-feature paradigm

Our results indicate that the fast, musical, multi-feature paradigm presenting deviants embedded in a complex musical pattern can elicit distinct MMNs not only in postlingually deaf adults but even in prelingually deaf adolescent CI users. Since MMNs are elicited pre-attentively with no behavioral task, this paradigm may be used for objective evaluation of CI users’ auditory skills in general and ability to discriminate musical sounds in particular. Because it is fast with a recording time of only 20 min and highly flexible with regard to both the nature and the deviation magnitude of the properties which it investigates, this paradigm could be a useful tool for assessing auditory rehabilitation following cochlear implantation. In a clinical context, MMN responses could be of relevance as an objective marker for measuring auditory discrimination abilities in CI patients, especially pediatric CI users, whose assessment of auditory discrimination and implant outcome is challenging. The paradigm does, however, run at a fast pace and a future revision should evaluate the effects of a reduced tempo, allowing analysis of effects in the 200–400 ms, particularly the P3a (Torppa et al., 2012).

### The impact of hearing age

The adolescent CI users in our study represented a huge range of age at implantation as well as communication background. Nevertheless, apart from the indication of an association between higher hearing age and shorter latencies for rhythm and saxophone, we found none of these factors predictive of either neurophysiological or behavioral performance. Especially with regard to the behavioral tests, this suggests that skills associated with cognition, concentration, attention, and memory may have a stronger impact than implant experience and prior use of sign language. As an interesting single case, CI 5, who is profoundly deaf, raised as a sign language user and who received his implant at the age of 9 years was able to score in the high average level of his group in both speech and music tests.

### Limitations

Recording and analyzing EEG with CI users represent a number of challenges. Due to the position of the implant, some electrodes cannot be used, resulting in a number of interpolated channels. Furthermore, due to the electric signal from the implant, it is necessary to use elaborate preprocessing procedures to reduce the CI artifact (Sandmann et al., 2009; Viola et al., 2011), allowing interpretation of the resulting evoked potentials of interest. Finally, in this particular study, recordings were done in the field, thus potentially degrading the signal-to-noise ratio as compared to recordings made in the shielded settings of the laboratory. In sum, these challenges may have resulted in data, which were less consistent than desired. Furthermore, measuring ERPs in a group of healthy individuals and a special group such as CI users implies an intrinsic difficulty of picking up the same peak for both groups. We cannot preclude that the applied peak-identification method, which identified MMN peaks algorithmically and separately in the two groups, erroneously may have led us to peaks from the two groups that in fact belonged to different ERP components.

The adolescent CI users in this study belong to the first generation of children who were offered CIs. Since, at the time, neo-natal hearing screening was not a standard procedure and some concerns about the safety of the surgery existed, they were in general both diagnosed and implanted later in childhood than is typical today. Therefore, they may not be fully representative of the future generations of early-implanted adolescents. We will, however, argue that the study and its findings are relevant, particularly considering the considerable number of teenagers worldwide making up this generation.

## Summary and Conclusion

Our findings provide novel insight on neural processing of musical sounds in a new generation of deaf adolescents, who have grown up with the assistance of CIs. The results showed that despite prelingual deafness and late implantation, adolescent CI users possess prerequisites for some discrimination of musical sounds, as indicated by their significant MMN responses particularly to changes in timbre, rhythm, and intensity. Compared to a NH reference, however, the CI users’ general discrimination abilities were characterized by significantly weaker brain responses and poorer behavioral performance. This was particularly true for their discrimination of small changes in pitch, which showed a severe deficit, reflected in inconsistent brain responses, and poor behavioral performance. Evidently, perception of music – especially melody – is degraded in these adolescent CI users, as also signified by the challenges observed in relation to singing. This, however, does not necessarily reduce music appreciation. Unlike postlingually deaf adult CI users, prelingually deaf CI users make no comparisons with previous music listening experience and may be quite satisfied with the representation provided by the implant, perceiving possibly particularly the rhythmic content of music (Gfeller et al., 2012). The lack of findings with an ear training program lasting only 2 weeks in CI users shows their refractoriness to auditory interventions. Thus, we encourage future research on the effects of longitudinal music training, preferably involving a combination of music making and training applications offering an adaptive and game-like interface. As observed here, the great compliance and enthusiasm of the participants indicate that such measures could be relatively easily implemented.

## Conflict of Interest Statement

The authors declare that the research was conducted in the absence of any commercial or financial relationships that could be construed as a potential conflict of interest. The Guest Associate Editor Teppo Särkämö declares that, despite being affiliated to the same institution as author Elvira Brattico, the review process was handled objectively and no conflict of interest exists.
